# Spatial coexistence of invasive ants in fragmented urban habitats of their native range

**DOI:** 10.3389/finsc.2026.1776153

**Published:** 2026-02-24

**Authors:** Ignacio J. Muñoz, Agustín Alvarez Costa, Pablo E. Schilman, Luis A. Calcaterra

**Affiliations:** 1 Laboratorio de Ecofisiología de Insectos, Facultad de Ciencias Exactas y Naturales, Departamento de Biodiversidad y Biología Experimental, Universidad de Buenos Aires, Buenos Aires, Argentina; 2 Instituto de Biodiversidad y Biología Experimental y Aplicada (IBBEA), CONICET-Universidad de Buenos Aires, Buenos Aires, Argentina

**Keywords:** community structure, ecological dominance, foraging behavior, resource monopolization, species interactions

## Abstract

**Introduction:**

Urban landscapes are increasingly recognized as key arenas for biological invasions, yet the mechanisms enabling the local coexistence of multiple highly invasive species remain poorly understood. Urban habitat fragmentation generates mosaics of habitat patches that differ in size, isolation, and microhabitat complexity, shaping ant community structure and competitive interactions.

**Methods:**

Here, we investigated ant assemblages across a mosaic of urban habitat patches within a university campus in Buenos Aires, Argentina, focusing on four globally invasive ant species (*Wasmannia auropunctata*, *Linepithema humile*, *Nylanderia fulva*, and *Solenopsis invicta*) near the southern limit of their native ranges. We quantified species richness, abundance and composition using pitfall traps and evaluated species-specific indicators of food discovery, recruitment, and dominance using standardized bait experiments.

**Results:**

Ant assemblages differed significantly among habitat patches, with marked spatial variation in richness, diversity, and species composition. Contrary to expectations of rigid dominance hierarchies, no single species consistently dominated across patches. *Nylanderia fulva* showed the highest numerical abundance and discovery efficiency, *L. humile* exhibited the strongest recruitment ability, and *W. auropunctata* displayed localized dominance near nesting areas, while *S. invicta* was rare and competitively subordinate. Ordination and multivariate analyses indicated strong spatial structuring of assemblages, consistent with the influence of urban fragmentation and patch-level heterogeneity.

**Discussion/Conclusion:**

Overall, our results support a metacommunity perspective in which invasive ant coexistence in urban systems is mediated by context-dependent competitive interactions rather than fixed dominance hierarchies. By emphasizing the role of fine-scale spatial structure, this study provides a nuanced, system-specific contribution to understanding invasive ant dynamics in urban environments.

## 1 Introduction

The structure of ant communities is fundamentally shaped by habitat heterogeneity and competitive interactions over limited resources. Urbanization, a major driver of habitat fragmentation, creates mosaics of habitat patches with altered microclimates, variable resource availability, and differing degrees of isolation, which can facilitate species coexistence by reducing direct resource overlap (1–4). These conditions often favor invasive ants, which are particularly successful in disturbed and heterogeneous environments (5–7), often dominating native assemblages (6–10), contributing urban biotic homogenization (11).

Urban green spaces (UGS), including reserves, parks, and tree-lined sidewalks, function as refuges within this fragmented matrix and can be conceptualized as habitat patches analogous to islands embedded in a hostile urban matrix (3, 12, 13). From this perspective, several complementary ecological frameworks predict patterns of species richness, composition, and coexistence. Island biogeography and habitat-based hypotheses predict higher richness in larger and less isolated patches, while the environmental heterogeneity hypothesis posits that increased microhabitat diversity promotes coexistence by reducing niche overlap and competitive exclusion (14). At broader spatial scales, metacommunity theory integrates these mechanisms by emphasizing the joint roles of environmental filtering, dispersal limitation, and patch connectivity (15).

In ant communities, competitive interactions over food and nesting sites often generate dominance hierarchies governed by species’ abilities to discover and monopolize resources, a relationship traditionally framed as the discovery–dominance trade-off (16, 17). However, resource monopolization is highly context-dependent, and habitat heterogeneity can weaken rigid dominance structures, allowing dynamic coexistence through spatial and temporal niche partitioning (18–22).

The local coexistence of multiple highly invasive ant species remains poorly documented, particularly in urban environments within their native ranges. *Wasmannia auropunctata*, *Linepithema humile*, *Solenopsis invicta*, and *Nylanderia fulva* are among the most globally invasive ants, yet their simultaneous occurrence is rarely reported. Each species exhibits distinctive ecological and behavioral traits influencing competitive outcomes, including differences in foraging speed, recruitment capacity, thermal tolerance, and nesting strategies (23–27). While studies in both native and invasive ranges demonstrate that competitive dominance among these species is strongly context-dependent and modulated by disturbance and habitat structure (26, 28–31), empirical data from highly fragmented urban landscapes remain scarce. This gap is particularly relevant given recent detections and predicted expansions of these species in urban areas worldwide.

From a theoretical perspective, urban habitat patches can be viewed as ecological “islands” embedded within a heterogeneous matrix, where dispersal limitation, environmental filtering, and local biotic interactions jointly shape community structure. Under this framework, island biogeography, environmental heterogeneity, and metacommunity theory generate the expectation that invasive ants with contrasting competitive strategies may coexist locally in fragmented urban systems. In this study, we use standardized field sampling and behavioral assays to evaluate how these predictions are reflected in ant assemblages across urban habitat patches. We ask whether fine-scale urban fragmentation and habitat heterogeneity can facilitate the local coexistence of multiple highly invasive ant species despite their strong competitive abilities.

This study aims to investigate patterns of coexistence and competitive interactions among four globally invasive ant species (*W. auropunctata*, *L. humile*, *S. invicta*, and *N. fulva*) in a fragmented urban landscape. Specifically, we (i) examine spatial variation in species richness and composition across urban habitat patches, (ii) compare species-specific indicators of discovery, recruitment, and dominance, and (iii) explore how fine-scale habitat heterogeneity and fragmentation influence local assemblage structure and coexistence. By focusing on a well-defined and intensively sampled urban system, this study provides a context-dependent assessment of invasive ant interactions, avoiding broad generalizations while offering insights relevant to urban invasion ecology. Because these species co-occur within their native range, this system provides a rare opportunity to disentangle intrinsic competitive strategies from invasion-related artifacts such as enemy release or evolutionary novelty.

## 2 Materials and methods

### 2.1 Study area

We conducted a field study to assess ant community structure and relative indicators of competitive ability among four highly invasive ant species that locally coexist in apparent sympatry at the southern limit of their distributions in Argentina. Sampling was carried out using a combination of pitfall traps and attractant baits following Calcaterra et al. (26, 29) and Chifflet and Calcaterra (32) during March and April 2021 on the grounds of the campus of the University of Buenos Aires (34°32′S, 58°26′W) in the northeastern of the city of Buenos Aires.

The region has a warm temperate to humid subtropical climate, with a mean annual temperature of approximately 17 °C and mean annual precipitation of 1236 mm, evenly distributed throughout the year (33). During summer, mean temperatures range between 20 and 25 °C, with average maximum temperatures frequently reaching 28–30 °C, while daily temperatures throughout the year typically fluctuate between 5 and 30 °C.

The campus covers an area of approximately 60 hectares and comprises a mosaic of built environments (e.g., buildings, parking lots, streets, and sidewalks) interspersed with green areas adjacent to the study sites (e.g., parks, soccer fields, and a natural reserve). Over time, the campus has undergone multiple structural and environmental modifications, including improvements in access to public spaces, the development of new public and private transportation networks, the extension of artificial lighting, and the expansion of green areas. The latter involved the incorporation of herbaceous vegetation and native tree species, resulting in a heterogeneous urban landscape with different vegetation strata. Despite ongoing human intervention, the campus and the adjacent reserve functions as an urban green space and potential coastal biological corridor, supporting diverse assemblages and generating fine-scale environmental heterogeneity relevant to ant nesting, foraging, and dispersal dynamics.

### 2.2 Pitfall trap sampling

**Figure 1:**
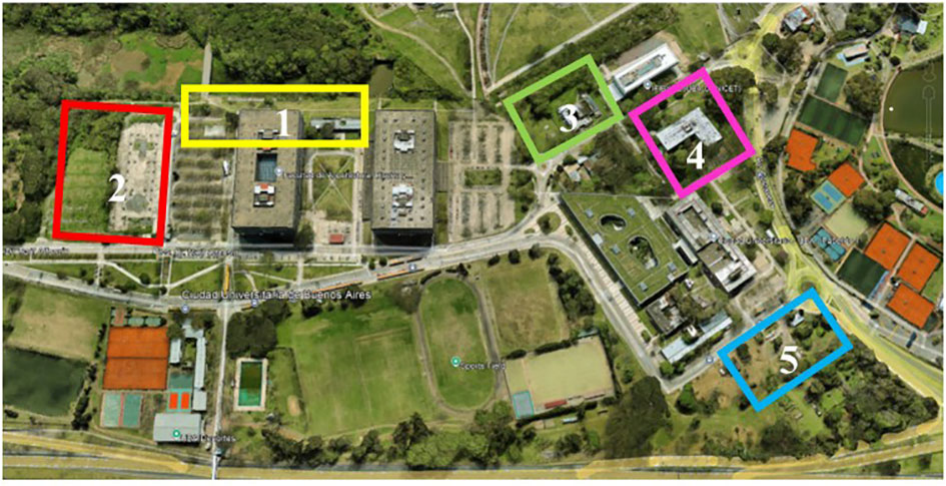
Study zones in the campus of the University of Buenos Aires: (1) Reserve, traps 1 to 26 (yellow); (2) Parking, traps 27 to 40 (red); (3) Animal facility, traps 41 to 52 (green); (4) Industries, traps 53 to 71 (pink); (5) Experimental field, traps 72 to 90 (light blue).

A total of 90 pitfall traps were set along transects distributed across five urbanized zones, where the ground surface was fragmented by buildings, streets, and paved paths, and vegetation varied in structural complexity and light availability. Pitfall traps consisted of 50 ml Falcon^®^ tubes buried at ground level and filled halfway with a solution of water and a few drops of detergent. They were spaced at least 10 m apart to minimize trap interference and spatial dependence among samples (34, 35). The five sampled areas were: (1) area next to the reserve, northeast of pavilion 3 (Reserve, traps 1 to 26); (2) surroundings of the parking lot, near the foundations of pavilion 4 (Parking, traps 27 to 40); (3) area of the animal facility located north of IFIBYNE (Animal facility, traps 41 to 52); (4) around the industries pavilion (Industries, traps 53 to 71); and (5) vicinity of the entrance to the experimental field, southeastern of pavilion 1 (Experimental field, traps 72 to 90) (**Figure 1**).

Traps were active for 48 hours, except for two that were lost. After removing the traps, their contents were emptied into Petri dishes, from where all representatives of the Formicidae family were separated and placed inside labeled Eppendorf tubes. The number of individuals of each morphospecies was separated and counted. Using a binocular microscope, and with the aid of available taxonomic keys and comparison with material deposited in the Fundación para el Estudio de Especies Invasivas (FuEDEI), Argentina, specimens were identified to genus or species when possible. Ant nomenclature follows Bolton et al. (36). Voucher specimens were deposited at the entomological collections of the FuEDEI.

Based on pitfall data, Spatial abundance (Sa) was calculated as the number of traps in which each species appeared, over the total number of traps. Numerical abundance (Na) was calculated as the number of individuals of a species or morphospecies of ant captured, over the total number of individuals captured of all species or morphospecies. Both abundance measures (spatial and numerical) were calculated as Calcaterra et al. (26). Species predominance (Sp) in pitfalls was also calculated as the percentage of traps in which each species has the greatest number of ants, as in Josens et al. (37) and Solá (38). Potential overrepresentation of highly recruiting species is not treated as a methodological artifact, but as an ecologically meaningful component of competitive performance. Because comparisons were made among species and zones using standardized sampling effort, differences in abundance reflect consistent interspecific differences in recruitment and foraging behavior rather than sampling bias.

### 2.3 Bait sampling

To study the dominance relationships within the ant assemblage, sampling with baits was carried out, using the same transects drawn in different zones of the university campus: reserve, parking area, animal facility, industries and experimental field (**Figure 1**). A total of 90 baits were deployed at the same locations as the 90 pitfall traps used in the previous survey. Each bait consisted of approximately half a spoonful of peanut paste (Bon-o-Bon) placed on a hard plastic square (5x5cm). As with the pitfall traps, baits were positioned at least 10 m apart. They were monitored over a 90-min period at 15-min intervals, recording the species or morphospecies present and the number of workers of each, except for the first measurement within the first 15 minutes, in which only the presence of the species or morphospecies that initially discovered the bait was recorded. If more than one species was present at this first observation, the event was considered a shared discovery and excluded from discovery index calculations to avoid bias.

### 2.4 Behavioral indices

With the different data obtained pitfall traps and baits, different behavioral indices were calculated that reflect the discovery, recruitment and dominance capabilities of each species. Bait incidence was calculated as the number of baits in which each species appeared over their total (Bi). With the percentage of baits that a species first discovered (Fd), and its spatial abundance in pitfalls (Sa), the ability to discover food (Df) for each species was calculated.

A null model was used in which all species are equally good discoverers by dividing the sum of Fd by the sum of Sa of all species recorded in the baits and multiplying by Sa of each species. The residual discovery (Rd) is obtained by subtracting this null model from the Bi of each species. This value gives an idea of how far each species is from the expected bait discovery value due to its abundance (that is why this index can acquire negative values).

To have a criterion about which species dominated each bait, the following parameters from Calcaterra et al. (29) were used: 1) A species dominates the bait if at least 5 workers are present for two consecutive observation periods (30 minutes), and in that period there are none or fewer workers of another species in the bait; 2) A species attempts to usurp the bait, if at least 3 workers are present on the bait for more than two observation periods (30 minutes); and 3) A species change occurs in the bait, if the usurper species displaces the one that was dominating it and retains it for at least two observation periods (30 minutes) with at least 5 own workers in the bait. The species that successfully usurped the bait becomes dominant.

Taking only the baits where a species was declared dominant and no other species were present in bait, the average number of workers recruited per species at the end of the trial (minute 90) was calculated, and this value was divided by the numerical abundance (Na) to obtain a mass recruitment index (MRi) independent of its abundance. Finally, three different dominance indices were calculated: spatial (Sd), numerical (Nd) and ecological (Ed). The first is equivalent to the spatial abundance (Sa), the second is equivalent to the numerical abundance (Na) in pitfalls, and the third one is the division between the number of dominated baits (Db) and the Sa. This last value reflects the ability of a species to dominate a resource, regardless of its abundance in the environment.


$$Df=F_{d}/S_{a}\;Rd=B_{i}-\left(\sum \;CF_{d}/\sum \;S_{a}\right).S_{a}\;Ed=D_{b}/S_{a}$$

### 2.5 Statistical analysis

To estimate asymptotic species richness while accounting for potential sampling bias and rare species, we calculated three widely used non-parametric estimators (Chao-1, iChao-1, and ACE). These estimators are sensitive to different aspects of rare species and sampling coverage, and their ensemble average was used as a robust indicator of expected total species richness inclusive of unobserved taxa (39).

Ant diversity was further assessed using three complementary diversity indices. The Shannon-Wiener Index (H) integrates species richness and abundance. The Evenness Index (J) measures the equitability of species abundances. The Simpson Index (1-D) emphasizes dominance patterns, providing a complementary perspective to Shannon diversity. The mean number of species captured per pitfall traps was compared among zones using analysis of variance (ANOVA), with area treated as a fixed factor. *Post-hoc* pairwise comparisons were conducted using Tukey honestly significant difference (HSD) test.

Differences in species composition among zones were explored using non-metric multidimensional scaling (NMDS) based on the Jaccard similarity index, applied to presence-absence data in pitfall traps. The Jaccard index was chosen to emphasize species turnover among patches independently of local abundance, which is often highly aggregated in ant assemblages. Exploratory analyses using abundance-based dissimilarities (Bray–Curtis) produced qualitatively similar spatial patterns. Because relatively high stress values are common in heterogeneous urban communities and in ordinations based on presence–absence data, NMDS results were interpreted as exploratory, whereas statistical inference relied primarily on pairwise analyses of similarities (ANOSIM).

To compare the proportions of baits discovered and dominated by different species, generalized linear mixed models (GLMMs) with a binomial error distribution were fitted. For each bait–species combination, discovery or domination was coded as presence (1) or absence (0). Species was included as a fixed effect, and bait was included as a random intercept to account for non-independence among observations. Model fit was evaluated by inspecting residual deviance relative to residual degrees of freedom to detect potential extra-binomial variation; no substantial overdispersion was evident after accounting for bait as a random effect. *Post-hoc* pairwise comparisons among species were performed using Tukey-adjusted contrasts. The relationship between species’ abilities to discover and dominate baits was evaluated using a Spearman rank correlation between the total numbers of baits discovered and dominated by each species.

Worker recruitment dynamics were analyzed using generalized linear mixed models (GLMMs) with worker abundance per bait as the response variable. Species was included as a fixed effect, and bait was included as a random intercept to account for repeated observations. The inclusion of bait as a random effect accounts for unmeasured heterogeneity among recruiting colonies, including variation in effective colony size. An initial model assuming a Poisson error distribution was fitted; however, model diagnostics indicated strong overdispersion, assessed by comparing the sum of squared Pearson residuals to the residual degrees of freedom (dispersion parameter > 1). To account for this overdispersion, models were refitted using a negative binomial distribution (nbinom2 parameterization). Model adequacy was subsequently evaluated through inspection of Pearson residuals versus fitted values and reassessment of the dispersion parameter, which fell within acceptable limits (≈1). Only baits occupied by a single species throughout the 90-minute observation period were included in the analysis. *Post-hoc* pairwise comparisons among species were performed using Tukey-adjusted contrasts. All statistical analyses were conducted using R (version 4.3.1; 40), with the packages lme4, glmmTMB, and emmeans.

## 3 Results

### 3.1 Species richness and abundance

A total of 6,690 worker ants representing 28 species across 18 genera were collected from the five sampled zones within the university campus (**Supplementary Table S1**). Species richness varied notably among zones, with the experimental field zone exhibiting the highest species numbers, and the industries zone the lowest (**Table 1**). Expected species’ richness also differed by zone, ranging from 17.4 species in the industries zone to 23.5 species in the parking zone, with the cumulative richness across all zones estimated at 31.5 species (**Table 1**). Mean species richness differed significantly among zones (F_4_, _83_ = 4.87; p< 0.0014), with the experimental field (6.11 ± 0.44) and industries (5.58 ± 0.43) (zones dominated by *N. fulva* and *L. humile*, respectively) exhibiting significantly higher richness than the parking zone, dominated by *W. auropunctata* (3.47 ± 0.48). The animal facility (5.08 ± 0.54) and reserve (4.63 ± 0.38) zones showed intermediate values. Diversity and evenness followed similar trends: the animal facility, where no single species dominated, had the highest diversity (1-D = 0.83; H = 2.08) and evenness (J = 0.72), while the experimental field zone, dominated by *N. fulva*, showed the lowest diversity (1-D = 0.40; H = 1.05) and evenness (J = 0.36).

**Table 1:** Diversity indices for species captured using pitfall traps in the five zones of the university Campus in the city of Buenos Aires, Argentina.

						All zones
	Reserve	Parking	Animal facility	Industries	Exp. field	
Expected species	19.09	23.53	21.25	17.40	19.17	31.46
Observed species	18	17	18	16	19	28
% Obs./Exp. spp.	94.27	72.25	84.71	91.97	99.10	89
Simpson (1-D)	0.71	0.44	0.83	0.58	0.40	0.84
Shannon-Wiener (H)	1.62	1.07	2.08	1.40	1.05	2.16
Evenness (J)	0.56	0.38	0.72	0.50	0.36	0.65

**Figure 2:**
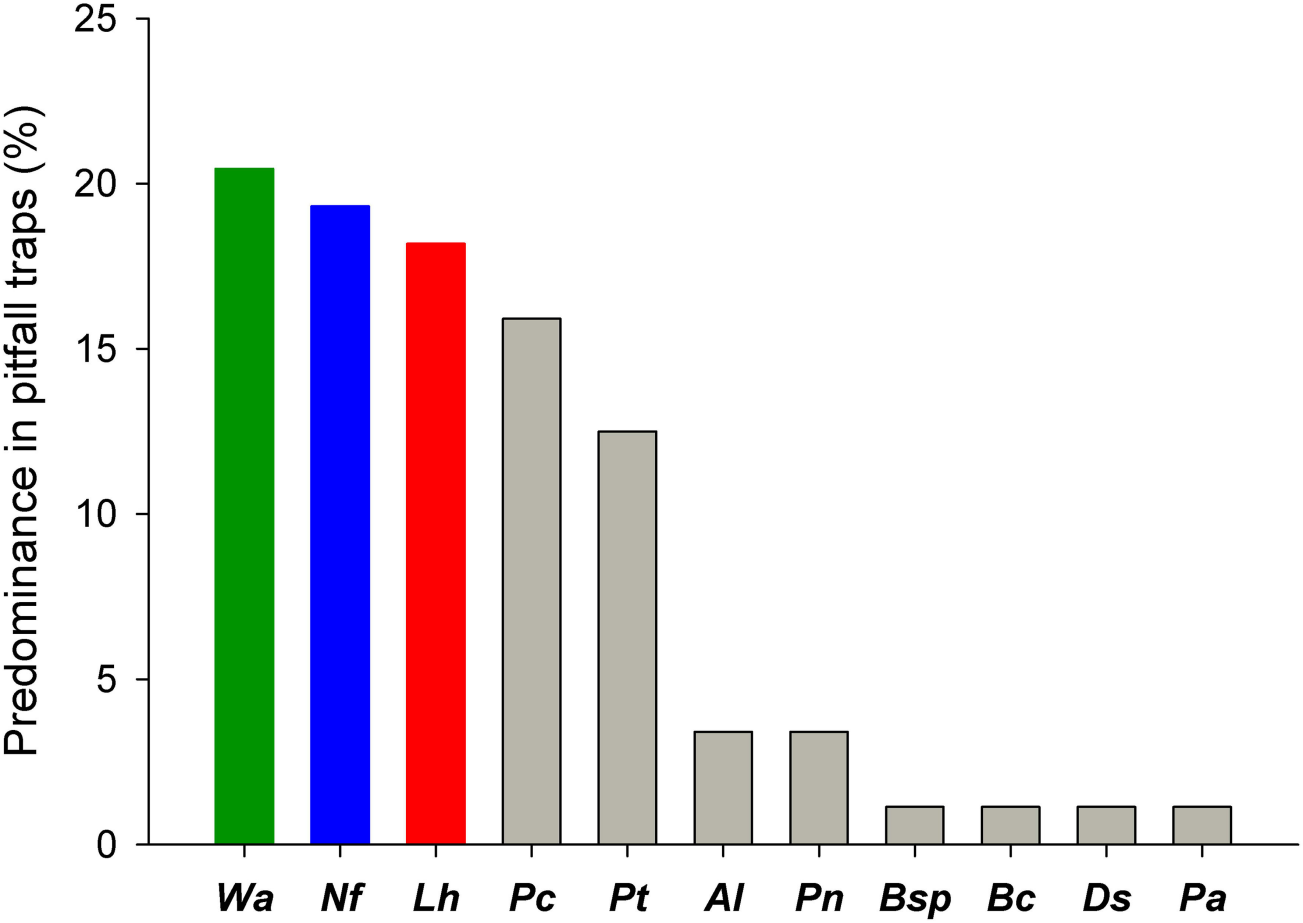
Percentage of pitfall traps with the highest number of individuals (predominance) per species during the pitfall trapping survey conducted on the university Campus: *Wasmannia auropunctata* (*Wa*), *Nylanderia fulva* (*Nf)*, *Linepithema humile* (*Lh*), *Pheidole cordiceps* (*Pc*), *Pheidole triconstricta* (*Pt*), *Acromyrmex lundii* (*Al*), *Pogonomyrmex naegeli* (*Pn*), *Brachymyrmex cordemoyi* (*Bc*), *Brachymyrmex* sp. (*B*sp), *Dorymyrmex steigeri* (*Ds*) and *Pheidole aberrans* (*Pa*). Color denotes worldwide invasive ant species.

The most spatially abundant species overall were *W. auropunctata* (50%) and *L. humile* (43.2%), followed by *N. fulva* (22.7%) and *S. invicta* (13.6%) (**Table 2**). Notably, *W. auropunctata* was highly frequent in two contiguous zones (reserve and parking) but never spatially dominated those assemblages due to its aggregated distribution. Conversely, *L. humile* and *N. fulva* were more spatially abundant in two other distinct zones (industries and experimental field, respectively). Numerically, *N. fulva* was the most abundant species (30.7%), followed by *Acromyrmex lundii* (14.6%), *L. humile* (13.9%), *W. auropunctata* (13.3%), and *S. invicta* (1.8%). However, *W. auropunctata* accounted for the highest proportion of traps with larger abundance (21.6%), followed by *N. fulva* (19.3%), *L. humile* (18.2%), and the native non-invasive *Pheidole cordiceps* (17.1%) (**Figure 2**).

**Table 2:** Ant species captured most frequently (>10% of traps) with pitfall traps and attracted to baits in the five study zones in the university campus. The table shows, for each species, the number and percentage of samples (pitfall traps) where it was found (spatial abundance), the total workers captured and their share of all workers (numerical abundance), plus the number of baits visited with the percentage of total baits (baits incidence) in parentheses.

Ant species	No. (%) samples	No. (%) workers	No. (%) baits
*Pheidole cordiceps* Santschi, 1923	48 (54.5)	473 (7.1)	40 (43.5)
*Solenopsis clytemnestra* Emery, 1895	45 (51.1)	236 (3.5)	19 (20.7)
***Wasmannia auropunctata* (Roger, 1863)**	**44 (50)**	**892 (13.3)**	**18 (19.6)**
***Linepithema humile* (Mayr, 1868)**	**38 (43.2)**	**927 (13.9)**	**31 (33.7)**
*Pheidole triconstricta* Forel, 1912	37 (42.1)	380 (5.7)	7 (7.6)
*Acromyrmex lundii* (Guérin-Méneville, 1838)	35 (39.8)	978 (14.6)	20 (21.7)
*Pogonomyrmex naegeli* Emery, 1895	29 (33)	174 (2.6)	–
*Brachymyrmex cordemoyi* Forel, 1895	22 (25)	71 (1.1)	14 (15.2)
*Brachymyrmex* sp. 1	20 (22.73)	95 (1.4)	–
***Nylanderia fulva* (Mayr, 1862)**	**20 (22.7)**	**2054 (30.7)**	**20 (21.7)**
*Cyphomyrmex rimosus* (Spinola, 1851)	19 (21.6)	50 (0.7)	–
*Solenopsis wasmanni* Emery, 1894	13 (14.77)	107 (1.6)	–
***Solenopsis invicta* Buren, 1972**	**12 (13.64)**	**122 (1.8)**	**8 (8.7)**
*Nylanderia steinheili* (Forel, 1893)	12 (13.64)	27 (0.4)	–
*Dorymyrmex steigeri* (Forel, 1914)	11 (12.5)	30 (0.4)	–

Invasive ant species are highlighted in bold.

### 3.2 Species composition

**Figure 3:**
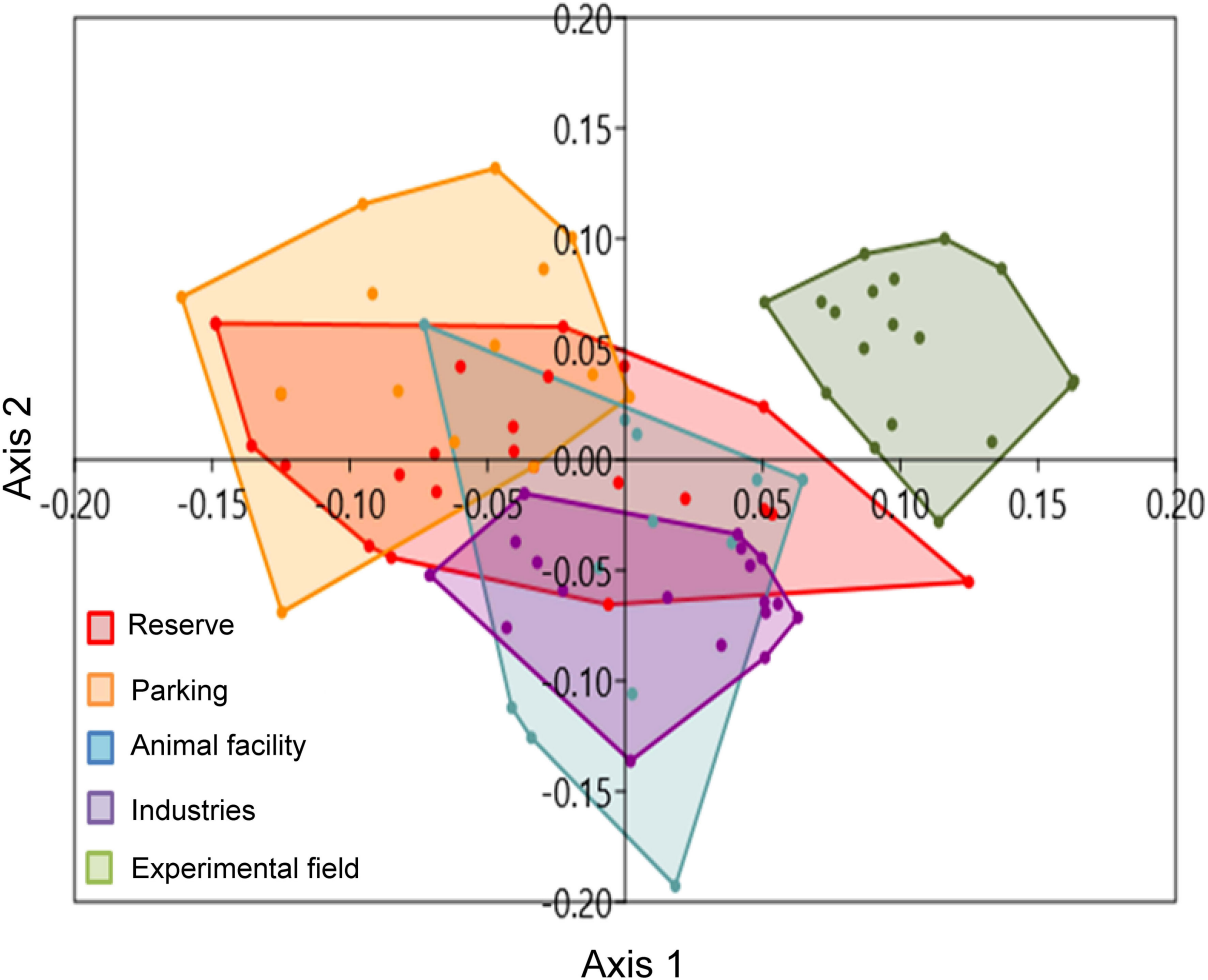
Non-metric multidimensional scaling analysis (NMS) using species presence data in pitfalls distributed in the five zones sampled in the university campus (R^2^ = 0.45; Stress = 0.3). Each cloud represents a different zone: Reserve (red), Parking (orange), Animal facility (blue), Industries (purple) and the Experimental field (green) zones.

NMDS ordination analyses revealed distinct differences in species composition among zones, with segregation particularly evident between the experimental field, parking, and animal facility zones (**Figure 3**; R² = 0.45, Stress = 0.3). Although stress was moderately high, indicating some limitation of the ordination, ANOSIM confirmed significant differences among zones (R = 0.45; p< 0.0001). Pairwise comparisons showed that the reserve zone differed significantly from the industries and experimental field zones, and that the parking zone differed from the animal facility, industries, and experimental field zones (p ≤ 0.04). No significant differences were found between the reserve and parking zones and between the reserve and animal facility zones, suggesting that assemblage differentiation could be positively associated with spatial distance or habitat fragmentation effects.

### 3.3 Discovery, recruitment and dominance

Only nine of the 28 species (32%) captured in pitfall traps were attracted to baits (**Table 2**). *P. cordyceps* had the highest bait incidence (43.3%), followed by *L. humile* (34.4%), *N. fulva* (22.2%), *W. auropunctata* (20%), and *S. invicta* (8.7%). Discovery rates differed significantly among species (χ²_8_ = 38.93; p< 0.001), with *N. fulva* (0.64), *S. invicta* (0.41), and *L. humile* (0.33) showing higher discovery indices, while *W. auropunctata* underperformed (0.20) (**Table 3**). When considering abundances, *N. fulva* (7.53), *L. humile* (2.61), and *S. invicta* (1.72) discovered more baits than expected, whereas *W. auropunctata* discovered fewer (-2.75) than expected (**Table 3**).

**Table 3:** Discovery, recruitment, and dominance abilities of the nine most common ant species in baited stations across five zones of the university campus measured using six indices: food discovery (Fd), residual discovery (Rd), workers recruited at 90 min/Na (MRi), spatial dominance (Sd), numerical dominance (Nd), and ecological dominance (Ed). Values are followed by the species’ rank (hierarchical order) in parentheses.

Species	Discovery		MRi	Dominance		
	Fd	Rd		Sd	Nd	Ed
***N. fulva***	**0.64 (1)**	**7.53 (2)**	**1.47 (3)**	**22.73 (8)**	**30.70 (1)**	**0.39 (1)**
***L. humile***	**0.33 (5)**	**2.61 (3)**	**2.27 (1)**	**43.18 (4)**	**13.86 (3)**	**0.33 (2)**
*P. cordyceps*	0.45 (2)	8.88 (1)	0.93 (4)	54.55 (1)	7.07 (5)	0.29 (3)
***W. auropunctata***	**0.20 (7)**	**-2.75 (7)**	**1.54 (2)**	**48.86 (3)**	**13.33 (4)**	**0.27 (4)**
*A. lundii*	0.28 (6)	0.43 (6)	**-**	39.77 (6)	14.62 (2)	0.11 (5)
*S. clytemnestra*	0.02 (9)	-11.3 (9)	–	51.14 (2)	3.53 (7)	0.07 (6)
*P. triconstricta*	0.03 (8)	-9.11 (8)	**-**	42.05 (5)	5.68 (6)	0.05 (7)
*B. cordemoyi*	0.36 (4)	1.99 (4)	–	25.00 (7)	1.06 (9)	0.00 (8)
***S. invicta***	**0.41 (3)**	**1.72 (5)**	**-**	**13.64 (9)**	**1.82 (8)**	**0.00 (9)**

Bold font indicates invasive ant species.

**Figure 4:**
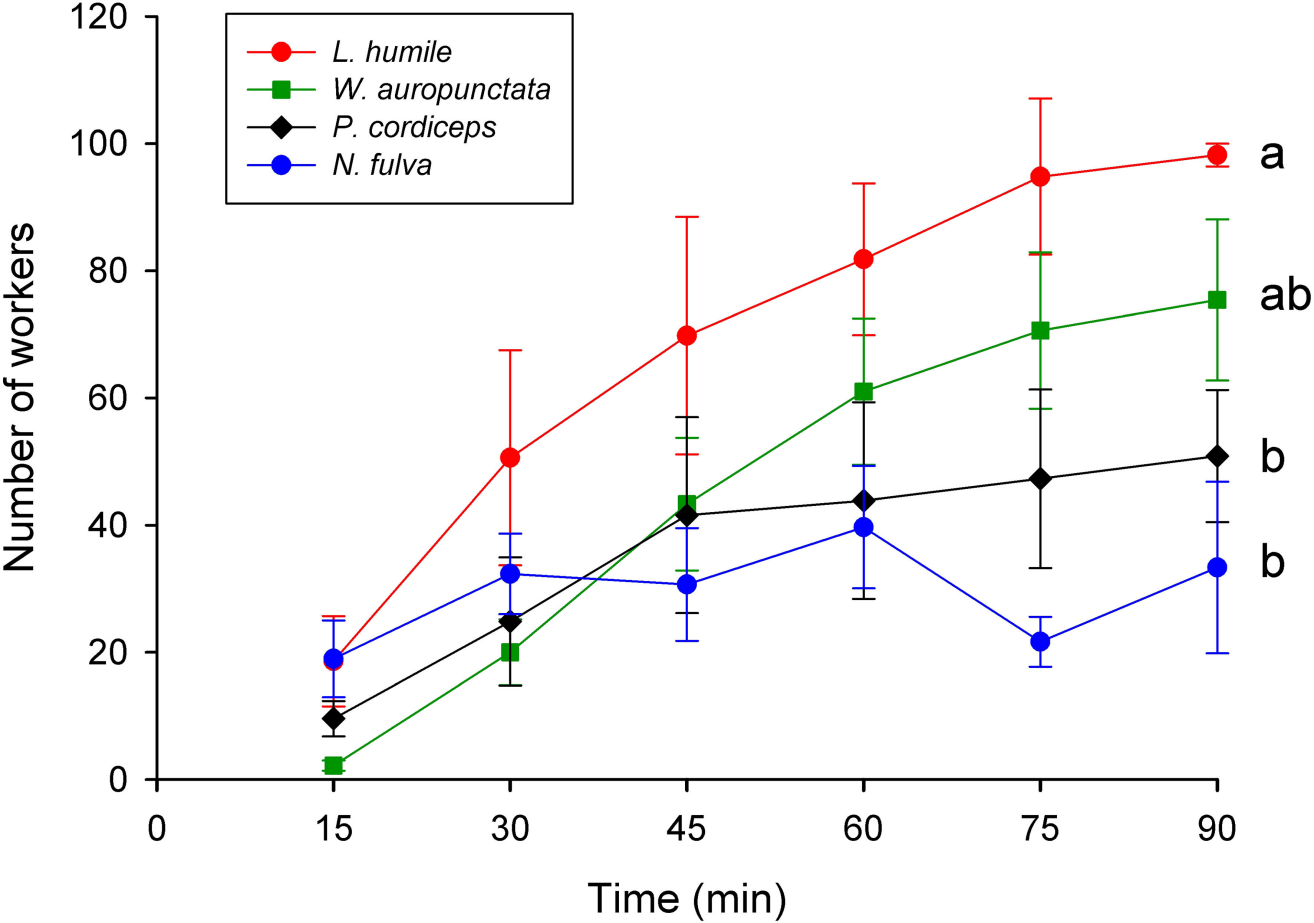
Mean (± SE) number of workers recruited to the baits every 15 min during the 90-minute trial: *Linepithema humile* (n = 5, red), *Wasmannia auropunctata* (n = 9, green), *Pheidole cordiceps* (n = 7, black), and *Nylanderia fulva* (n = 3, light blue). Means ± standard errors are shown for the six observation periods. Different letters denote significant differences (p< 0.05). Color denotes worldwide invasive ant species.

Recruitment patterns differed significantly among the four species analyzed (χ²_3_ = 17.21; p = 0.0006), with *L. humile* recruiting significantly more workers per bait (~98 workers over 90 minutes) than *P. cordyceps* (~51 workers, *post-hoc* p = 0.0024) and *N. fulva* (~33 workers, *post-hoc* p = 0.0043), (**Figure 4**); though in the case of *N. fulva* only three baits could be included in this analysis. Other species that recruited many workers at baits were excluded for the same reason. Considering their numerical abundance (Na), the three invasive ant species ranked higher in their mass recruitment index than *P. cordyceps* (**Table 3**).

**Figure 5:**
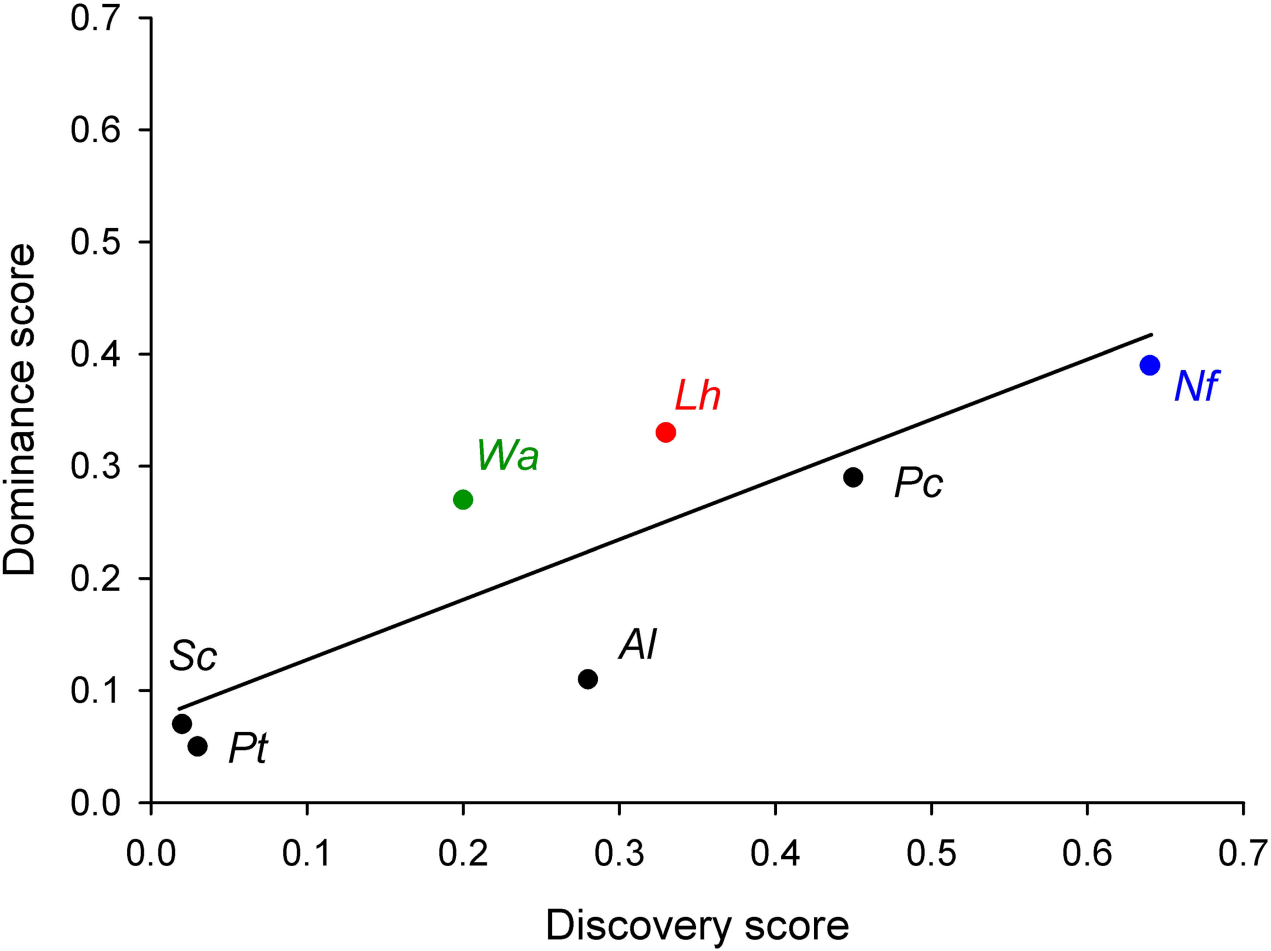
Relationship between the abilities to locate (Food discovery) and dominate (Ecological dominance) baits (r_s_ = 0.89, p = 0.013) placed on the grounds of the university campus for the seven most attracted ant species: *Pheidole triconstricta* (*Pt*), *Solenopsis clytemnestra* (*Sc*), *Wasmannia auropunctata* (*Wa*), *Acromyrmex lundii* (*Al*), *Linepithema humile* (*Lh*), *Nylanderia fulva* (*Nf*), *Pheidole cordiceps* (*P_C_*). Color denotes worldwide invasive ant species.

Spatial dominance (Sd) was highest for *P. cordyceps* (54.6%) and *Solenopsis clytemnestra* (51.1%), whereas numerical dominance (Nd) was highest for *N. fulva* (30.7%) and *Acromyrmex lundii* (14.6%) (**Table 3**). Ecological dominance (Ed), an abundance-independent metric, was highest for *N. fulva* (0.39) and *L. humile* (0.33). A strong positive correlation was detected between the number of baits discovered and the number dominated by each species (Spearman r_s_ = 0.89, p = 0.0135; **Figure 5**).

## 4 Discussion

This study examined patterns of coexistence and competitive interactions among four globally invasive ants (*W. auropunctata*, *L. humile*, *N. fulva*, and *S. invicta*) within a fragmented urban landscape located near the southern limit of their native ranges. By combining measures of species richness and abundance and assemblage composition, and species-specific indicators of discovery, recruitment, and dominance, our results reveal strong spatial structuring of ant assemblages and pronounced interspecific differences in competitive strategies across urban habitat patches. Together, these findings directly address our initial question by showing that fine-scale spatial heterogeneity within urban landscapes can facilitate the local coexistence of multiple highly invasive ant species, even in the absence of a single, consistently dominant competitor. Overall our results support the view that local coexistence among invasive ants in urban systems is context dependent and mediated by fine-scale spatial heterogeneity rather than by rigid dominance hierarchies.

### 4.1 Effects of urban fragmentation on assemblage structure

Species richness, abundance and composition varied markedly among the five zones sampled within the university campus. Zones characterized by lower numerical dominance of a single species exhibited higher diversity and evenness, whereas zones dominated numerically by one invasive species showed reduced evenness despite comparable richness. This pattern indicates that urban fragmentation modulates not only species richness but also the internal structure of assemblages, generating communities that may be similarly rich yet sharply differ in dominance and equitability. Accordingly, richness alone is a poor descriptor of assemblage structure in invaded urban systems, where dominance effects strongly shape evenness. This pattern is also consistent with predictions from island biogeography and environmental heterogeneity hypotheses, which posit that patch-level differences in size, isolation, and microhabitat structure can strongly influence local diversity and community composition (1, 3, 11, 12).

Although habitat variables were not quantified directly, the spatial segregation of assemblages detected by NMDS and supported by ANOSIM suggests that urban habitat patches within the campus function as semi-independent units embedded within a heterogeneous urban matrix (3). While urban environments are often associated with biotic homogenization driven by the replacement of specialists by widespread generalists (11), our results suggest that heterogeneity within the urban matrix can maintain distinct assemblages at fine spatial scales. Together, these patterns indicate that urbanization does not necessarily lead to complete biotic homogenization at local scales, as the urban matrix itself can actively mediate dispersal, colonization, and competitive interactions among habitat patches (41). Variation in impervious surface cover, vegetation structure, and connectivity, likely alters matrix permeability, thereby differentially facilitating movement, nesting, and foraging for invasive ant species with contrasting ecological traits (5, 7, 42).

Comparisons with previous surveys in Buenos Aires and surrounding semi-natural habitats indicate that fragmented urban mosaics disproportionately favor a small subset of invasive ants, whereas more continuous habitats support richer and more even assemblages (26, 37). In this context, the urban matrix may act simultaneously as a barrier and a filter, constraining movement for some species, while enhancing connectivity for others (43), thereby shaping metacommunity dynamics and diversity patterns across habitat patches (2, 15).

### 4.2 Species-specific competitive strategies

The four focal invasive ants exhibited contrasting combinations of discovery, recruitment, and dominance, underscoring the absence of a single, universally dominant species across the urban landscape, as commonly reported for ant communities where competitive outcomes are strongly context dependent (22). This outcome is consistent with our expectation that competitive hierarchies in fragmented urban systems are contingent on local context rather than species identity alone. Instead, competitive outcomes appear to be mediated by species-specific strategies that differentially balance rapid resource discovery, recruitment efficiency, and numerical dominance. Among focal species, *N. fulva* emerged as the most numerically dominant and most efficient resource discoverer, consistent with its rapid spread and competitive impacts reported in other systems (44). Its high residual discovery values indicate an ability to exploit resources rapidly beyond what would be expected from its spatial abundance alone, suggesting that fast exploration and recruitment initiation are key components of its competitive strategy. Recent reports from comparable urban environments, including an university campus in Costa Rica, further support the role of *N. fulva* as a dominant invader capable of rapidly restructuring local ant assemblages (45). Although currently dominant at the campus scale, its long-term persistence and landscape-level dominance are likely to depend on local environmental conditions and patch connectivity, rather than reflecting a universally superior competitive ability.

In contrast, *L. humile* exhibited the highest recruitment rates at baits, rapidly monopolizing resources. This pattern is consistent with its well-documented mass-recruitment behavior and unicolonial social structure, which enable large worker mobilization and efficient resource exploitation (8, 46). However, despite its strong recruitment ability, *L. humile* did not dominate numerically across all zones, underscoring the importance of local environmental constraints, such as habitat fragmentation and microclimatic variability, in limiting its spatial expansion, particularly near the edge of its native range (26, 47, 48).

*Wasmannia auropunctata* showed moderate spatial and numerical abundance but relatively low food discovery ability, consistent with previous studies describing it as a poor discoverer, likely reflecting constraints imposed by its smaller body size and slower foraging speed, with dominance often restricted to areas near nesting sites (24, 32). Nevertheless, once established at a bait, *W. auropunctata* was able to achieve intermediate levels of dominance, suggesting that localized recruitment and aggressive defense can compensate for slower discovery in patchy environments. This spatially restricted dominance likely contributes to its coexistence with other invasive ants by limiting direct interference competition over larger spatial scales (49, 50).

*Solenopsis invicta* was rare and showed negligible dominance within the sampled zones, a pattern that contrasts sharply with its strong ecological dominance in warmer regions. Its low abundance and limited competitive impact here likely reflect a combination of climatic constraints, restricted suitable open habitat, and recent colonization near the southern edge of its range (25). This finding reinforces the importance of regional and local context in determining invasion success and competitive outcomes.

### 4.3 Context-dependent dominance

The pronounced interspecific differences in discovery, recruitment, and dominance argue against a purely neutral assembly process and instead point to strong niche-based mechanisms structuring ant assemblages in the urban landscape. Although discovery and dominance were positively correlated across species, high discovery did not invariably translate into high ecological dominance, as illustrated by the contrasting strategies of *N. fulva* and *W. auropunctata*. These results suggest that competitive outcomes depend not only on intrinsic species traits but also on local environmental context and spatial structure. The positive correlation between discovery and dominance may reflect processes operating at the scale of individual patches, whereas at broader spatial scales dominance appears to become decoupled from discovery due to fragmentation and limited inter-patch connectivity.

Rather than conforming to a classical discovery–dominance trade-off, invasive ants in this system exhibit distinct, context-dependent strategies assumed to be shaped by habitat heterogeneity (21, 30). In such heterogeneous urban mosaics, variation in patch size, isolation, and matrix permeability may favor species that combine rapid resource discovery with flexible recruitment responses, while constraining species that rely more heavily on territorial dominance. As a result, spatial niche partitioning likely weakens rigid dominance hierarchies and facilitates coexistence within our fragmented urban mosaic, consistent with growing evidence that discovery–dominance patterns are highly contingent and often weak or absent in heterogeneous environments (10, 21, 22).

Within this framework, *N. fulva* stands out as the numerically dominant and most efficient resource discoverer on the university campus, reflecting its rapid invasion dynamics and increasing prevalence in urban environments. Its marked rise in abundance and strong capacity for resource discovery, especially in fragmented urban landscapes, underscore its potential as a major invasive threat. Importantly, the dominance of *N. fulva* may be contributing to a shift in competitive hierarchies, potentially reducing the prevalence of the *L. humile* super colony previously reported for the campus experimental zone (38). Such dynamics highlight that dominance relationships among invasive ants are not static, but can be restructured over relatively short temporal scales as novel invaders establish and expand.

### 4.4 Implications for invasion ecology in urban systems

Although this study was conducted within a single urban site and over a restricted sampling period, our results highlight general mechanisms by which urban fragmentation and fine-scale habitat heterogeneity can structure invasive ant assemblages. Urban mosaics may promote spatial niche partitioning and limit the spatial extent of competitive exclusion, thereby allowing different invasive species to dominate locally under specific environmental contexts (18, 19). In this framework, the ongoing spread of *S. invicta* in southern Europe provides a relevant case for evaluating how habitat heterogeneity and fragmentation may influence interactions with previously established invasive ants, such as *L. humile* or *W. auropunctata*, even in a climate change context (31, 51, 52).

Although *N. fulva* is currently restricted to the Americas, its biological traits and invasion success suggest a high potential for introduction and establishment in tropical and subtropical urban regions worldwide, including Europe, Asia, and Oceania (44, 53, 54). Understanding its competitive dynamics and spatial niche partitioning is therefore essential for anticipating future invasion scenarios involving co-occurring invasive ants. Importantly, behavioral interactions observed within native ranges may differ in invaded regions, where altered competitive environments, enemy release, and climatic conditions can modify species interactions. Accordingly, caution is warranted when extrapolating these findings to invaded urban systems or management contexts beyond the study area.

From a management perspective, our results indicate that urban habitat configuration and microhabitat diversity can modulate invasion dynamics by shaping connectivity and local competitive outcomes. Management actions that alter habitat structure may therefore have context-dependent effects on invasive ant assemblages, underscoring the need to integrate landscape structure and species-specific traits when designing strategies for urban invasion management (55, 56).

## 5 Conclusions

This study shows that multiple globally invasive ant species can coexist locally in fragmented urban habitats when competitive interactions are mediated by fine-scale spatial heterogeneity. In such landscapes, invasive ants exhibit contrasting and partially decoupled strategies of resource discovery, recruitment, and dominance, preventing the establishment of rigid dominance hierarchies. These results suggest that as invasive ant species become increasingly introduced into the same regions, their local co-occurrence is likely to be structured by spatial heterogeneity and context-dependent interactions, rather than leading to the exclusion of all but a single dominant invader. By explicitly linking spatial heterogeneity to context-dependent competitive interactions, this study challenges the expectation of inevitable competitive exclusion among dominant invaders and highlights the potential of fragmented urban landscapes to sustain the coexistence of multiple highly invasive ants, even within their native range.

## Data Availability

The raw data supporting the conclusions of this article will be made available by the authors, without undue reservation.
