# Supplementary material for: Spatial coexistence of invasive ants in fragmented urban habitats of their native range
Source: Front Insect Sci. 2026 Feb 24;6:1776153. doi: 10.3389/finsc.2026.1776153 (PMC12971952; doi:10.3389/finsc.2026.1776153)
Supplement: Supplementary file 1 [file DataSheet1.pdf]

Supplementary Material

Supplementary Table S1. Ant species captured using pitfall traps (samples) in the five sampled zones of the university campus in the city of Buenos Aires.

Species	No. captured workers of each species (% occupied samples)					
	Reserve<br>n = 24	Parking<br>n = 15	A. facility<br>n = 12	Industries<br>n = 19	Exp. field<br>n = 18	Total<br>n = 88
Pheidole cordiceps	210 (63)	53 (40)	79 (75)	17 (32)	114 (67)	473 (55)
Solenopsis clytemnestra	24 (33)	7 (20)	32 (50)	34 (63)	139 (89)	236 (51)
Wasmannia auropunctata	432 (71)	435 (100)	4 (33)	21 (42)	0	892 (50)
Linepithema humile	83 (38)	3 (6.7)	94 (75)	747 (100)	0	927 (43)
Pheidole triconstricta	31 (21)	48 (13)	77 (67)	178 (90)	46 (28)	380 (42)
Acromyrmex lundii	872 (50)	17 (40)	17 (33)	54 (75)	18 (22)	978 (40)
Pogonomyrmex naegeli	128 (63)	12 (27)	24 (33)	7 (26)	3 (6)	174 (33)
Brachymyrmex cordemoyi	7 (25)	2 (13)	1 (8)	55 (42)	6 (22)	71 (25)
Brachymyrmex sp.	6 (8)	1 (7)	5 (17)	49 (42)	34 (33)	95 (23)
Nylanderia fulva	0	2 (13)	1 (8)	0	2051 (94)	2054 (22.7)
Cyphomyrmex rimosus	7 (21)	2 (13)	1 (8)	0	40 (61)	50 (22)
Solenopsis wasmanni	4 (8)	0	5 (17)	4 (16)	94 (33)	107 (15)
Solenopsis invicta	24 (4)	0	0	0	98 (61)	122 (14)
Nylanderia steinheili	2 (8)	3 (20)	0	10 (16)	12 (22)	27 (14)
Dorymyrmex steigeri	23 (25)	0	2 (13)	0	5 (20)	30 (13)
Pheidole aberrans	6 (8)	0	12 (13)	3 (5)	2 (5)	23 (7)
Pheidole flavens	0	0	0	8 (16)	6 (16)	14 (7)
Strumygenis louisianae	0	1 (7)	2 (17)	0	2 (11)	5 (6)
Hypoponera opaciceps	1 (4)	0	3 (8)	0	2 (11)	6 (5)
Pheidole bergi	5 (8)	0	7 (8)	0	0	12 (3)
Gnaptogenys triangularis	1 (4)	1 (7)	0	1 (5)	0	3 (3)
Crematogaster sp.	0	1 (7)	0	0	1 (6)	2 (2)
Dorymyrmex brunneus	0	0	1 (8)	0	0	1 (1)
Hypoponera opacior	0	0	0	3 (5)	0	3 (1)
Camponotus punctulatus	0	1 (7)	0	0	0	1 (1)
Pseudomyrmex termitarius	0	1 (7)	0	0	0	1 (1)
Typhlomyrmex pillosus	0	0	0	1 (5)	0	1 (1)
Neivamyrmex sp.	0	0	0	0	2 (6)	2 (1)
Total number workers	1866	590	367	1192	2675	6690

Supplementary Figures

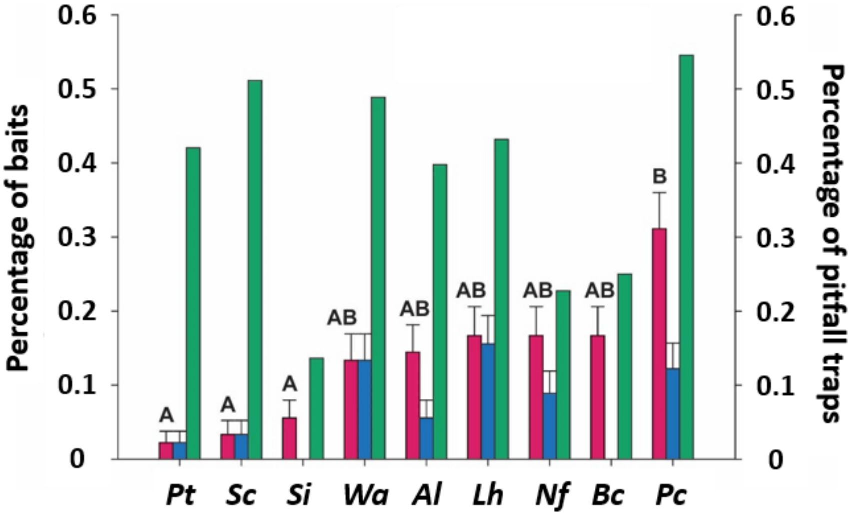

Supplementary Figure 1. Proportion of baits discovered (in red) and dominated (in blue) by each species across five zones of the university campus: *Pheidole triconstricta* (*Pt*), *Solenopsis clytemnestra* (*Sc*), *Solenopsis invicta* (*Si*), *Wasmannia auropunctata* (*Wa*), *Acromyrmex lundii* (*Al*), *Linepithema humile* (*Lh*), *N. fulva* (*Nf*), *B. cordemoyi* (*Bc*), and *Pheidole cordiceps* (*Pc*). Probabilities are shown with their standard errors. Frequency of each species in pitfalls (in green) is included for comparison. Different letters indicate significant differences ($p < 0.05$).
